# Impacts of Compost Bedded Pack Barns on the Welfare and Comfort of Dairy Cows

**DOI:** 10.3390/ani10030431

**Published:** 2020-03-04

**Authors:** Anna Fernández, Eva Mainau, Xavier Manteca, Adriana Siurana, Lorena Castillejos

**Affiliations:** Animal Nutrition and Welfare Service, Department of Animal and Food Science, Facultat de Veterinària, Universitat Autònoma de Barcelona, Bellaterra, 08193 Barcelona, Spain; annafernandezpi@gmail.com (A.F.); eva.mainau@uab.cat (E.M.); xavier.manteca@uab.cat (X.M.); adrisiuri@gmail.com (A.S.)

**Keywords:** compost bedded pack barn, lying behavior, integument alteration, cow dirtiness, cow comfort

## Abstract

**Simple Summary:**

As few studies have examined the most common housing systems in terms of cow behavior and welfare, we designed this study to compare three loose housing systems for lactating cows: compost bedded pack, conventional bedded pack, and freestalls. Cows kept in bedded pack barns were dirtier yet had fewer hairless patches and lesions or swellings on the body and spent more time lying down in the resting area than cows housed in freestalls. All housing systems show benefits and inconveniences in terms of welfare and comfort at resting. Other factors, such as management practices and proper barn design, should be considered when comparing housing systems in terms of animal welfare.

**Abstract:**

Three loose housing systems for lactating cows (compost bedded pack, CBP; conventional bedded pack, BP; and freestalls, FS) were assessed on one farm in terms of cow behavior and welfare. An on-farm welfare assessment based on the Welfare Quality protocols was used four times every three months on 757 cows. Video recordings taken twice over four days were used to assess behavior patterns at resting areas. Cows in CBP and BP were dirtier than those in FS (*p* < 0.0001). Fewer integument alterations were recorded for CBP and BP than FS (*p* < 0.001). Cows in BP were quicker to lie down and stand up compared to those in CBP or FS (*p* < 0.001). Percentages of cows needing more attempts before rising were higher for FS (*p* < 0.01). However, a higher frequency of kneeling was observed in CBP (*p* = 0.033). A lower percentage of cows lying in the resting area was recorded for FS (56%) than CBP or BP (97 or 84%, respectively, *p* < 0.05). Overall, in this study, cows kept in bedded pack barns were dirtier but had fewer integument alterations and spent more time lying down in the resting area than cows housed in freestalls.

## 1. Introduction

The assessment of cow comfort on dairy farms is essential for strategies aimed at maintaining welfare, health, and production rates, or even to increase these rates. To properly assess animal welfare, Fraser [1] and Webster [2] stressed the need for measures based on both the environment and the animal. In fact, studies have shown that housing and management have a major impact on the overall welfare of dairy cows [3]. 

Loose housing systems allow dairy cows to move freely around the barn and to adopt natural behavior patterns. Haley et al. [4] emphasized the potential impact of housing facilities on lying behavior. Lying has been described as the highest priority behavior when compared with relative priorities such as eating, social contact and other behaviors [5]. Reduced lying time because of uncomfortable flooring triggers behavioral and physiological stress responses that may affect the cow’s health and performance [6]. Both the layout and the type and quality of the bedding material are crucial to maintain adequate cow comfort. Uncomfortable barns in which cows spend a shorter time lying will mean a longer time standing in concrete corridors, increasing the risk of claw diseases and injuries [7,8]. Consistent with this idea, [9] observed a higher prevalence of claw lesions in dairy cows housed in freestalls compared with compost bedded pack barns.

A compost bedded pack barn is an alternative loose housing system that has recently gained popularity. According to Barberg et al. [10], producers opt for this type of housing system as cow comfort, health, and longevity seem improved and daily chores are easier. Compost bedded pack barns have been described as effective dairy housing systems in terms of lying behavior, social interactions and natural lying positions [11]. The conventional bedded packs and freestalls widely used today to house dairy cows have several limitations. The most important issues to be improved are the compromised cleanliness of conventional bedded packs, which has been linked to mastitis [12,13], and a high prevalence of claw lesions among dairy cows kept in freestall barns [9].

So far, a small number of studies have examined the effects of compost bedded pack barns on the health, behavior and welfare of dairy cows [10,11]. In another study, Barberg et al. [14] compared welfare, performance and udder health before and after changing the housing system in the same dairy barn. More recently, Husfeldt and Endres [15] assessed associations between different stall bedding materials and some animal welfare measurements in 34 dairy farms where 45% of the herds had mattresses and 55% had deep-bedded stalls. However, although some studies have started to compare composted beddings with other systems and beddings, we still lack data for comparisons among the three type of housing systems discussed on this paper obtained on the same farm and over the same time period. Our objective in this study was thus to examine cow comfort and behavior in lactating dairy cows kept in three different types of loose housing systems on the same farm: compost bedded pack (CBP), conventional bedded pack (BP) and freestalls (FS). To assess these two cow factors, we used the Welfare Quality assessment protocol for dairy cattle [16] and examined behavior patterns at resting areas. 

## 2. Materials and Methods 

### 2.1. Animals, Housing and Feeding

All methods involving cows were approved by the ethics committee of the Autonomous University of Barcelona (CEEAH-2734) and the Government of Catalonia (Spain) (DMAH-8240). This study was conducted at a dairy farm (Girona, Spain) over the period November 2013 to July 2014. The farm had 819 Friesian lactating cows kept in three different types of loose housing systems over the study period. In compliance with farm management, cows were randomly allocated to one of the barns for the entire lactation period irrespective of their parity or milk production. Cows were milked three times a day (0500, 1300 and 2100 h). The average milk yield per cow was 37.7 L/d, resulting in a total farm milk production of 30,909 L/d. Cows were fed TMR twice daily to give a daily feed consumption of 23.5 kg of dry matter per animal. Access to water was ad libitum. 

### 2.2. Treatments

Treatments (CBP, BP and FS) were allocated in two barns each. Freestalls had concrete flooring covered with a rubber mat. This treatment consisted of a double row of face-to-face freestalls in the middle of the barn. Stall dimensions were: total length of double face-to-face stalls 4.5 m; distance from rear of curb to rear of brisket locator (resting area) 1.65 m; center-to-center stall divider (stall width) 1.18 m; and height of neck rail above top of curb 1.03 m. For both bedded pack treatments (CBP and BP), 0.7 kg/m^2^ and 1.5 kg/m^2^ of dry sawdust were directly placed on the bedded pack surface once daily while cows were milked. Compost bedded pack barns were aerated twice a day to refresh the surface and promote microbial activity in the pack. The first aeration was performed just before the addition of bedding material. The bedded pack area was completely cleaned out at least every six months. For the BP barns, the bedded pack surface was not aerated, and the area was completed cleaned out at least every month. 

### 2.3. Data Collection and Study Design

All planned visits were notified to the manager beforehand to avoid disturbing the farm management program (including veterinary procedures and routine hoof trimming) and to make sure keepers were informed about being video recorded. The study design was divided into: (1) on-farm assessment and (2) behavioral assessment. 

On-farm assessment was conducted every 3 months by two trained observers (four times in total) To determine inter-observer reliability, 112 cows were assessed by both observers 1 week before starting the experiment. Before each assessment, observers reviewed all the measures included in the on-farm assessment protocol to ensure maximum agreement between them. Six barns (two barns per treatment) were examined in every visit. In total, 757 cows (256 cows assigned to CBP, 250 to BP and 251 to FS) were assessed during the experimental period. Before data collection, the sample size per treatment was calculated following the sampling recommendations for clinical scoring specified in the Welfare Quality assessment protocol for dairy cattle [16]. The number of animals indicated to score per treatment was proportionally split into two barns for each treatment. The animal-based measures assessed are described in Table 1. Assessment was performed on one side of the cows’ bodies randomly selected and balanced between observers in each visit.

Behavioral variables were assessed in recorded videos. Three barns (one barn per treatment) were filmed using two static video cameras (model IP66, Circontrol, Terrassa, Spain) per barn and two previously installed digital video recorders (model VDVR-4NX, Circontrol, Terrassa, Spain). Dimensions of the video-recorded area and video-recorded resting area are shown in Table 2. 

Cows were recorded on 4 consecutive days, 1 week after the second and third on-farm assessment visits. Each day, videos were obtained of two 3 h periods after the first and the second milking (starting 1 h after milking) and watched by a single trained observer. For intra-observer reliability, the same observer evaluated 3 h recordings for each treatment on two separate occasions. Behavior patterns observed by focal sampling and scan sampling at 5-min intervals are described in Table 3 and Table 4 respectively. 

### 2.4. Statistical Analysis

All statistical tests were performed using the Statistical Analyses System package (SAS V9.4; software SAS Institute Inc., Cary, NC, USA; 2002-2012). Significance was set at *p* < 0.05.

Inter-observer reliability was calculated for the on-farm assessment measures, and intra-observer reliability for the behavior assessment measures. For continuous variables, we used the Spearman correlation coefficient and for categorical variables the Kappa coefficient. The guidelines of Landis and Kock [20] were used to interpret the kappa test according to the grading system (<0.00 = poor, 0.00-0.20 = slight, 0.21-0.40 = fair, 0.41-0.60 =moderate, 0.61-0.80 = substantial, and 0.81-1.00 = almost perfect agreement). 

A normality test of data and residuals was performed for every on-farm and behavior measure and analyzed using generalized linear mixed models ( GLIMMIX procedure). The LSMEANS adjusted to Tukey’s honestly significant difference was used as a test of comparisons. 

For the on-farm assessment, dichotomous variables (scored as 0 or 2) were expressed as the number of animals with a score of 2 out of the total number of animals assessed. For other measures (scored as 0, 1 or 2) data were expressed as the number of animals with a score of 1 or 2 out of the total number of animals assessed. All variables followed a binary distribution. The model included the fixed effect of treatment (CBP, BP and FS) and stocking density as a covariate. Barn was the experimental unit. Barn and number of visits and observers were included as random effects in the model. 

The time taken for a cow to lie down or stand (analyzed as the average of time for each 3 h periods observations) followed a normal distribution after log transformation. For behavior measures determined for the lying and rising sequences, the dichotomous variables attempting the motion before its start, incorrect or unfinished motion and kneeling followed a binomial distribution. Percentages of animals lying or standing in the resting area followed a normal distribution. Other behavioral measures observed by scan sampling followed a Poisson distribution. The model included the fixed effect of treatment (CBP, BP and FS) and stocking density as a covariate. Barn was the experimental unit and number of visits was included as a random effect. 

## 3. Results and Discussion

### 3.1. Inter and Intra-Observer Reliability

Cohen’s kappa estimate ranged from 0.72 to 0.98 for the on-farm assessment measures, indicating a substantial and almost perfect inter-observer agreement. 

Intra-observer Spearman correlations for behavior patterns assessed by scan sampling ranged from 0.96 to 1 (*p* < 0.001 in all cases), except for behavior categories involving head position which ranged from 0.70 (*p* = 0.036) to 0.95 (*p* < 0.001). Measures of the time needed to stand up or lie down showed correlations of 0.82 (*p* < 0.001) and 0.60 (*p* = 0.038) respectively. Cohen’s Kappa for other behaviors assessed by focal sampling was 1.00 (perfect agreement).

### 3.2. On-Farm Assessment Measures

The impacts of treatment on the on-farm assessment measures are provided in Table 5.

#### 3.2.1. Body Condition

We found proportions of very lean and very fat cows was unaffected by treatment. Adams et al. [21] detected no relationship between body condition score and housing, and reported that the use of sprinklers and misters to cool cows and balanced feed rations reduced the percentage of thin cows. The cows examined here were subjected to similar cooling systems (fans) and rations. 

#### 3.2.2. Cow Dirtiness

Percentages of cows with dirty lower hind legs and dirty teats were unaffected by treatment. However, we did note higher proportions of cows with dirty udders among the CBP and BP housed animals than among those kept in FS (p < 0.001). Moreover, cows in BP showed the highest rate of hindquarter dirtiness, whereas cows in FS showed the lowest (p < 0.001 in all cases). Our observations are in line with those of Fregonesi and Leaver [13] who reported that cows housed in conventional bedded pack barns were dirtier than those kept in freestalls. A higher prevalence of dirtiness has also been described for compost housing systems compared with freestalls. For instance, Lobeck et al. [22] obtained higher hygiene scores for cows allocated to compost bedded pack barns than for cows allocated to either cross-ventilated or natural-ventilated free stall barns. However, others have highlighted the importance of compost management to ensure the proper quality of compost, which was found to be related to cow hygiene scores [23]. Cow hygiene is associated with mastitis risk and some studies have revealed that conventional housing systems show a positive relationship with mastitis incidence [12,13]. Although we have no data regarding the prevalence of mastitis and did not assess compost quality in our study, compost quality is a known important factor to reduce the average level of somatic cell count along with the incidence of clinical mastitis [14,24,25]. 

#### 3.2.3. Integument Alterations

Cows kept in FS showed a higher prevalence of moderate and severe integument alterations than those housed in CBP or BP (*p* < 0.001). When we examined integument alterations by body region, the following were unaffected by treatment: severe alterations in the tarsus, and moderate and severe alterations in the neck/shoulder/back, flank/side/udder and carpus. Greater percentages of cows with moderate (*p* = 0.035) and severe (*p* = 0.006) integument alterations in the hindquarters were detected for the FS than BP treatments. Cows in FS showed the highest rates of moderate integument alterations of the tarsus (*p* < 0.001), whereas cows in CBP showed the lowest (*p* = 0.007). Inconsistent with the cleanliness benefits reported for freestalls, our data point to more integument alterations (moderate and severe) in cows kept in FS compared to CBP or BP. Integument alterations reflect compromised welfare as wounds and swellings may be painful, and hairless patches suggest a lack of adaptation to the environment [26]. In general, freestalls have been associated with a high prevalence of lesions [27]. Effectively, Barberg et al. [14] reported that cows housed in compost bedded pack barns had fewer hock lesions when compared with data from other studies for freestall housing [27]. Similarly, van Gastelen et al. [28] and Husfeldt and Endres [15] noted a decreasing percentage of cows with healthy hocks with the hardness of the bedded surface. 

It should be noted that our FS treatment group showed higher integument alteration rates than those reported elsewhere [29], especially in the tarsus region. The freestalls in our study were smaller than those indicated in current recommendations for adult cows [30]. In fact, when freestalls are too short and/or the flooring is too hard, alterations of the hock and carpus are frequent because of the high momentum needed for the cows to lie down [31]. 

#### 3.2.4. Hoof Health and Lameness

Percentages of cows with overgrown hooves were unaffected by treatment. A higher proportion of cows showed inflamed coronary bands (at the top of the hooves) in the CBP treatment group than in FS (*p* = 0.025). Although we observed similar proportions of cows that were moderately lame across the treatment groups, the prevalence of severely lame cows was higher in CBP and BP than in FS (*p* = 0.029 and *p* = 0.033 respectively). Some authors have linked uncomfortable housing systems and a hard lying surface to a greater risk of injuries and lameness [27,32]. Although Lobeck et al. [22] reported a lower incidence of lameness among cows housed in compost bedded packs compared to cows housed in freestall sand barns, other studies [9,33] failed to show a difference in lameness between both systems. Discrepancies may be, in part, a consequence of the multiple factors and interactions associated with lameness or simply due to differences among housing systems and associated management practices [34]. 

### 3.3. Behavioral Assessment Measures

#### 3.3.1. Lying down and Standing up

Measures related to lying and standing are shown in Table 6. The number of events to lie down and stand up were analyzed as the average of time for each 3-h period of observation. 

The time needed to lie down from a standing position was longer in CBP and FS than in BP (*p* = 0.020) whereas the time needed for a cow to stand up was longer in FS compared to CBP and BP (*p* < 0.001) and shorter in BP compared with CBP (*p* < 0.001). According to Chaplin and Munksgaard [35], changing position from lying to standing can be restricted in free stalls by factors such as limited lounging space due to inappropriate stall design and dimensions. 

The longer standing/lying times we observed in CBP may be attributed to compost quality as its compaction hampers position changes. Effective composting requires maintaining an adequate temperature and moisture content through proper CBP handling [33].

The cows in our study allocated to CBP kneeled more often during the lying down process than those in BP (*p* = 0.033) or FS (*p* = 0.032). An increased kneeling response has been observed in cows when there is some obstruction to the normal lying down sequence [36]. According to prior results, we expected cows kept in freestalls to show increased kneeling times. However, it seems that our cows preferred to kneel first on the soft, comfortable compost bedding pack before lying down.

We also recorded the attempts made at lying and rising before finally embarking on the motion. More cows in FS made an attempt or several attempts before lying down than in BP (*p* = 0.006) and more cows did so before standing up in FS than in both CBP (*p* < 0.001) and BP (*p* < 0.001). An inappropriate stall design and hard lying surfaces may restrict normal behavior such that cows prepare more for their descending or ascending movement [32]. A hard floor surface may also explain a cow’s hesitance to initiate a position change. 

Other factors assessed here such as an incorrect or unfinished lying or rising motion or kneeling during the ascent motion were unaffected by treatment. 

#### 3.3.2. Posture and Position in the Barn

Behavioral measures related to the cows’ posture and position in the barn are provided in Table 7. 

When considering the percentage of cows lying in the resting area (as opposed to standing, walking or perching) we found that more cows did this in CBP and BP than FS (*p* = 0.012 and *p* < 0.001, respectively). Consistently, some studies have linked concrete flooring and uncomfortable stalls with an increased cow standing time [8,37]. 

For all three treatments, a head up position when lying was more common than head back, head flat on side or head on the ground, although rates of this head position were lower in FS than CBP and BP (*p* = 0.016 and *p* = 0.003, respectively). While we lack data to define cow comfort in terms of preferred head positions when lying, the three treatments allowed for all natural head positions (head back, head up, head flat on side, and head on the ground). Others have reported that housing type may influence lying down positions adopted by cows [11], especially in uncomfortable stalls, which may reduce the lying time due to the impossibility to adopt certain resting postures [4]. 

## 4. Conclusions

In the present study, cows kept in conventional bedded pack were generally dirtier than cows housed in compost bedded pack followed by cows in freestalls. Moreover, cows kept in both bedded packs spent more time lying down in the resting area and had fewer moderate and severe integument alterations than cows housed in freestalls. However, cows in conventional bedded pack were quicker to lie down and stand up compared to those housed in compost bedded pack and freestalls. Our finding suggests that cows kept in bedded pack barns were dirtier but had fewer integument alterations and spent more time lying down in the resting area than cows housed in freestalls. Other factors such as management practices and proper barn design should be considered when comparing housing systems in terms of animal welfare. 

## Figures and Tables

**Table 1:** Data collected in the on-farm assessment.

Measure	Description ^1^
Body condition	Four regions assessed: cavity around the tail head, loin, vertebrae and tail head/hipbones/spine/ribs. Score: Regular body condition (score 0), very lean (score 1) and very fat (score 2). The last two scores were recorded when at least 3 of 4 regions were very lean or very fat, respectively.
Dirtiness	Four regions assessed: lower hind leg, hindquarter, udder and teats. Scores: 0 = clean, 2 = dirty. When assessing lower hind leg, hind quarter and udder, *dirty* was defined as separate or continuous areas of dirt amounting to the size of the palm of the hand. Minor splashing on the teats was also considered dirty (score of 2).
Integument alterations	Hairless patches and lesions/swellings at least 2 cm diameter assessed in 5 regions: neck/shoulder/back, hindquarter, tarsus, flank/side/udder and carpus. Scores: 0 = no alteration, 1 = moderate alteration, 2 = severe alteration Hoof coronary band also included when assessing integument alterations. Scores: 0 = no alteration, 2 = coronary band swollen and reddish.
Hoof overgrowth	Hooves assessed assuming a normal length of 7-8 cm Score: 0 = 2 claw horns of one leg similarly normal in length, 2 = at least one claw horn of one leg too long.
Lameness	Three gait factors assessed: timing of steps, temporal rhythm and weight-bearing on feet. Scores: 0 = not lame, 1 = lame, 2 = severely lame.

^1^ All measures based on the Welfare Quality assessment protocol for cattle [16], except hoof overgrowth that was adapted from Archer et al. [17].

**Table 2:** Descriptive variables recorded over the study period in the compost bedded pack barns, bedded pack barns and freestalls.

Item ^1^	Treatment ^2^					
	CBP1	CBP2	BP1	BP2	FS1	FS2
No. of cows, mean	104.8	72.8	88.0	91.0	96.0	95.0
No. of cows, SE	2.93	1.49	2.45	2.87	0.00	0.50
Stocking density, mean	10.7	9.2	7.6	7.6	9.4	9.1
Stocking density, SE	0.32	0.18	0.21	0.25	0.00	0.50
Total area, m^2^	906	864	1121	695	670	695
Total resting area, m^2^	254	265	177	177	265	265
Video-recorded area, m^2^	181	NA	147	NA	280	NA
Video-recorded resting area, m^2^	116	NA	58	NA	190	NA

^1^ SE: standard error. ^2^ CBP1: compost bedded pack barn (barn 1); CBP2: compost bedded pack barn (barn 2); BP1: bedded pack barn (barn 1); BP2: bedded pack barn (barn 2); FS1: freestalls (barn 1); FS2: freestalls (barn 2); NA: not applicable.

**Table 3:** Ethogram of behaviors assessed by focal sampling.

Item	Description
Time needed to lie down, s	The event starts when one carpal joint of the animal is clearly bent (before touching the ground). The whole lying down sequence ends when the hindquarter has descended and the animal has moved the scapula or leg of the supported underside forward (adapted from Plesch et al. [18]).
Time needed to stand up, s	The event starts when the animal starts lifting the hindquarter from the ground. The rising sequence ends when both front legs touch the ground and the animal stands with the entire body weight on all 4 legs [18].
Cows undergoing an attempt/s before starting the motion, %	The lying down/standing up motion is preceded by one or more attempts at lying down or rising. The cow performs at least one attempt to: (1) lie down; when the carpal joint is clearly bent (before touching the ground) but, rather than continuing with the motion, the leg returns to its normal standing position; (2) stand up; when the hindquarter clearly moves forward and body weight leans on both knees but, rather than continuing with the motion, the animal moves backwards and remains in the lying position.
Incorrect or unfinished sequence, %	The lying down and rising sequence (described above) is incorrectly performed or unfinished.
Kneeling, %	Animal with knees bent supporting mainly body weight and the hind legs extended for at least 5 s (adapted from Krohn and Munksgaard, [19]).

**Table 4:** Ethogram of behaviors assessed by scan sampling at 5-min intervals over two 3h periods.

Item	Description
Lying in the resting area	
Lying head resting	Animal lying in the resting area with the head in a relaxed position in contact with the floor, housing equipment or its own body (but not turned backwards) (adapted from Plesch et al. [18]).
Lying head upright	Animal lying on the sternum in resting area with the head raised off the ground [4].
Lying head back	Animal lying on the sternum in resting area with the head turned backwards resting on the body [19].
Lying on side	Animal lying in resting area on its side with entire body weight on one side and legs not underneath the body [18].
Standing or walking in the resting area	Animal standing or walking in the resting area either with all 4 legs or perching (forelegs in resting area and hind legs in alley).

**Table 5:** Impacts of compost bedded pack barn, bedded pack barn and freestalls on cow welfare.

Item	Severity and Body Region	Treatment ^1^			SEM ^2^
		CBP	BP	FS	
No. of cows assessed		256	250	251	NA
Body condition	Very lean, %	4.6	4.2	2.3	1.68
	Very fat, %	19.0	12.7	8.4	5.27
Dirtiness	Lower hind leg, %	99.5	99.8	99.2	1.26
	Hindquarter, %	40.5 ^b^	53.2 ^a^	30.9 ^c^	3.37
	Udder, %	41.3 ^a^	44.2 ^a^	30.0 ^b^	2.86
	Teats, %	53.5	61.0	34.6	10.80
Integument alterations	Moderate (neck/shoulder/back), %	7.7	7.5	9.0	2.26
	Severe (neck/shoulder/back), %	0.1	0.3	0.1	0.96
	Moderate (hindquarters), %	22.5 ^a,b^	18.2 ^b^	25.3 ^a^	3.21
	Severe (hindquarters), %	5.0 ^a,b^	2.6 ^b^	13.1 ^a^	4.15
	Moderate (tarsus), %	2.0 ^c^	8.4 ^b^	62.0 ^a^	7.47
	Severe (tarsus), %	5.8	1.8	3.7	4.40
	Moderate (flank/side/udder), %	7.3	6.7	8.5	4.38
	Severe (flank/side/udder), %	2.6	3.4	2.4	2.52
	Moderate (carpus), %	0.3	2.0	2.9	3.04
	Severe (carpus), %	0.3	0.5	0.6	0.78
	Moderate (all body regions), %	34.4 ^b^	37.1 ^b^	72.3 ^a^	5.84
	Severe (all body regions), %	16.4 ^b^	16.0 ^b^	54.3 ^a^	15.01
Hoof health and lameness	Inflamed coronary band, %	15.1 ^a^	8.0 ^a,b^	0.6 ^b^	6.53
	Hooves overgrown, %	19.1	16.0	15.2	3.91
	Moderately lame, %	13.2	8.4	15.4	5.12
	Severely lame, %	4.2 ^a^	3.4 ^a^	0.7 ^b^	2.51

^1^ CBP: compost bedded pack barn; BP: bedded pack barn; FS: freestalls. ^2^ SEM: standard error of the mean; NA: not applicable. ^a–c^ Least square means within a row with different superscripts differ (*p* < 0.05).

**Table 6:** Effects of the treatments (compost bedded pack barn, bedded pack barn and freestalls) on each behavioral variable measured during the motion of lying down/standing up.

Item		Treatment ^1^			SEM ^2^
		CBP	BP	FS	
Lying down					
	No. of events observed	347	318	429	NA
	Time needed to lie down, s	4.9 ^a^	4.5 ^b^	4.9 ^a^	0.37
	Attempts before starting the motion, %	4.7 ^a,b^	1.5 ^b^	6.4 ^a^	3.32
	Incorrect or unfinished motion, %	2.3	0.6	1.9	0.53
	Kneeling, %	6.4 ^a^	2.2 ^b^	2.6 ^b^	1.32
Standing up					
	No. of events observed	251	356	357	NA
	Time needed to stand up, s	3.5 ^b^	3.1 ^c^	3.9 ^a^	0.30
	Attempts before starting the motion, %	3.3 ^b^	1.7 ^b^	14.9 ^a^	2.85
	Incorrect or unfinished motion, %	1.5	0.3	1.1	0.53
	Kneeling, %	0.8	0.6	1.9	0.73

^1^ CBP: compost bedded pack barn; BP: bedded pack barn; FS: freestalls. ^2^ SEM: standard error of the mean; NA: not applicable. ^a–c^ Least square means within a row with different superscripts differ (*p* < 0.05).

**Table 7:** Effects of the treatments (compost bedded pack barn, bedded pack barn and freestalls) on behavior patterns observed by scan sampling at 5-min intervals over two 3 h periods.

Item, % Time Over 6h		Treatment ^1^			SEM ^2^
		CBP	BP	FS	
Lying in the resting area		96.5 ^a^	84.5 ^a^	56.4 ^b^	9.17
	Head resting	1.3	1.1	2.1	2.03
	Head up	91.6 ^a^	91.8 ^a^	85.9 ^b^	5.46
	Head back	6.1	7.0	10.7	4.45
	Lying on side	2.9	0.2	3.8	3.75
Standing in the resting area		3.5 ^b^	15.5 ^b^	43.6 ^a^	9.17

^1^ CBP: compost bedded pack barn; BP: bedded pack barn; FS: freestalls. ^2^ SEM: standard error of the mean. ^a,b^ Least square means within a row with different superscripts differ (*p* < 0.05).
